# Treatment durability and conceptual validity of an innovative PT program targeting mobility among older Veterans

**DOI:** 10.1093/geroni/igaf122.4035

**Published:** 2025-12-31

**Authors:** Jonathan Bean, Rebekah Harris, Elisa Ogawa, Rachel Ward, Mary Kate Palleschi, Mariana Wingood, Thomas Travison, Jennifer Brach

**Affiliations:** Harvard Medical School, Boston, Massachusetts, United States; VA Boston Healthcare System, Boston, Massachusetts, United States; VA Boston Healthcare System, Boston, Massachusetts, United States; VA Boston Healthcare System, Boston, Massachusetts, United States; Albany Medical College, Albany, New York, United States; Wake Forest University School of Medicine, Winston-Salem, North Carolina, United States; Hebrew SeniorLife & Harvard Medical School, Hebrew SeniorLife, Massachusetts, United States; University of Pittsburgh, Pittsburgh, Pennsylvania, United States

## Abstract

We investigated the treatment durability and conceptual validity of an innovative physical therapy program known as Live Long Walk Strong (LLWS). LLWS is designed to enhance mobility by targeting leg power, trunk muscle endurance, gait variability and self-efficacy for exercise, which are all attributes linked to mobility decline. This was a longitudinal study of LLWS participants derived from a randomized controlled trial (RCT). The RCT participants in an 8-week waitlist control group crossed over to LLWS treatment. The combined cohort was then evaluated after treatment and over 16 weeks of subsequent follow up. This study was conducted at an outpatient VA tertiary care center among community-dwelling middle aged and older Veterans with slow walking speed. LLWS is an 8-week 10 (1-hour/session) session outpatient physical therapy program. Mobility measures included walking speed and the Short Physical Performance Battery (SPPB). We also evaluated the attributes LLWS targets. Within robust multivariable linear mixed effect models, we observed significant improvements (p<.05) in walking speed by ≥.09m/s after treatment and this magnitude of difference persisted 8 and 16 weeks after treatment ended. This pattern of improvement was also observed for the SPPB and all four attributes. Within stratified analyses, the initial treatment response on mobility surpassed a clinically meaningful threshold after adjusting for health factors commonly mitigating rehabilitative gains. Our study demonstrates that LLWS treatment effects are robust and sustained up to 4 months after treatment. As conceptually designed, LLWS produces large and prolonged improvements in measures of mobility and the attributes it targets.

